# Food intake precipitates seizures in temporal lobe epilepsy

**DOI:** 10.1038/s41598-021-96106-z

**Published:** 2021-08-13

**Authors:** Dalma Tényi, József Janszky, Sára Jeges, Andreas Schulze-Bonhage

**Affiliations:** 1 Department of Neurology, Medical School, University of Pécs, Rét u. 2, Pecs, 7623 Hungary; 2 Institute of Nursing and Patients Care, Faculty of Health Sciences, University of Pécs, Pecs, Hungary; 3 Epilepsy Center, University of Freiburg, Breisacher Str. 64, 79106 Freiburg, Germany

**Keywords:** Neurology, Epilepsy

## Abstract

Various factors have been considered as potential seizure precipitants. We here assessed the temporal association of food intake and seizure occurrence, and characteristics of seizures and epilepsy syndromes involved. 596 seizures from 100 consecutive patients undergoing long-term video-EEG monitoring were analyzed. Preictal periods of 60 min were assessed as to the occurrence of food intake, and latencies between food intake and seizure onset were analyzed. Seizures of temporal origin were highly significantly more frequently preceded by food intake compared to those of extratemporal origin; and were associated with shorter food intake-seizure latency. Seizure precipitation by food intake showed male predominance. Shorter food intake-seizure latency was associated with less severe seizures and less frequent contralateral spread of epileptic discharges. We here show for the first time that not only in specific rare reflex epilepsies but in the most frequent form of focal epilepsy, temporal lobe epilepsy, seizures are significantly precipitated by food intake. Seizure occurrence was increased over a period of up to one hour following food intake, and remained more localized in terms of both ictal EEG spread and as reflected by seizure severity. This finding supports the emerging concepts of ictogenesis, implying a continuum between reflex and spontaneous seizures—instead a dichotomy between them.

## Introduction

Epilepsy has classically been characterized by the occurrence of unprovoked and spontaneous seizures. Accordingly, the uncertainty and the constant fear of having a seizure were considered as a major contributor to quality of life impairments in people with epilepsy^1^. Recent studies have suggested that there are both, endogenous rhythms influencing seizure probability^2,3^ and a number of external factors claimed by patients to precipitate be seizure precipitants. Overall, patients can rate the probability of seizure occurrence above chance^4^. So far, the validity of individual seizure precipitants has, however, remained unclear, and both, recall bias and subjective wishes for causal attribution may contribute to retrospective patient attribution of seizure triggers^5^.

We here studied continuous video-EEG recordings in epilepsy patients to objectively assess temporal relationships between food intake and seizure occurrence in a large cohort of epilepsy patients to study food intake with its act of chewing and activation of the autonomic nervous system and the generation of focal-onset seizures. Furthermore, we assessed the exact temporal relationships and seizure characteristics, including seizure origin, severity and spread in seizures occurring following food intake vs. those occurring independently. Results are discussed in the context of new seizure classifications and the changing concepts of “reflex” vs. “unprovoked” seizures.

## Methods

**Figure 1:**
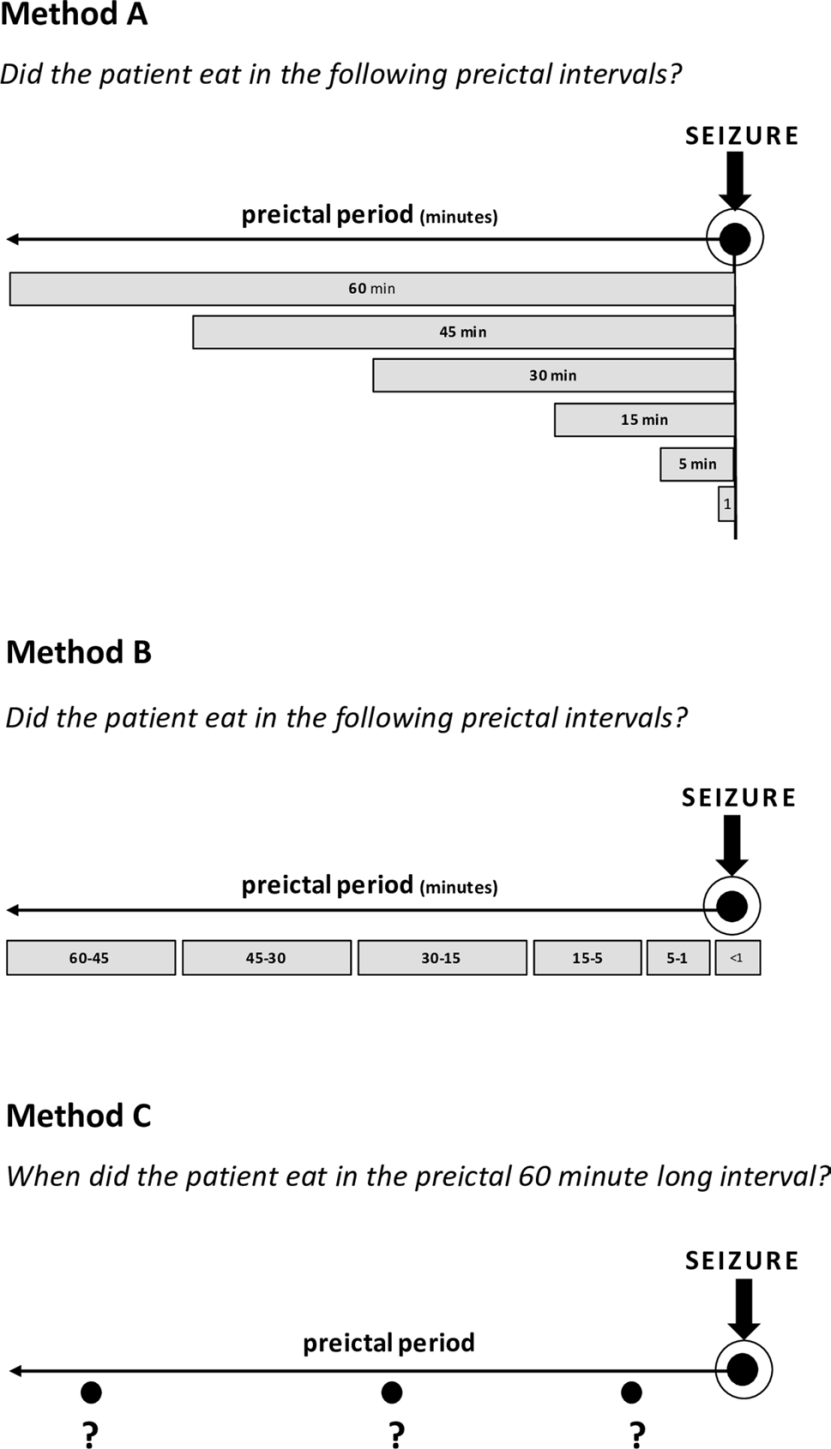
Principle of the analysis of the preictal period.

100 consecutive patients undergoing continuous long-term video-EEG monitoring in the years 2012 to 2017 at the Epilepsy Center, University of Freiburg, Germany were analyzed. From each patient, all or the first 10 recorded seizures were included in the statistical evaluation. EEG data were acquired using video-EEG systems from IT-med (Usingen, Germany). EEG recordings were performed with the application of surface electrodes according to the international 10–20 system, analyzing 21 channels, including T1/T2. When necessary, sphenoidal electrodes were also applied, as well as additional bilateral 10–10 electrodes in the suspected areas of seizure generation. EEG signals were low pass filtered at 1.6 Hz and high pass filtered at 70 Hz. Additional notch filter was also applied, eliminating the 50 Hz line noise. Preictal periods of 60 min were visually analyzed based on continuous video-EEG recordings to exactly define and time-stamp periods of food intake termination and the latency to the onset of the following seizures. Any act was considered as food intake if both chewing and swallowing solid consistency could be observed regardless of the amount of food. Intake of medication of use or chewing gum were not considered as food intake. The preictal 1 h was analyzed with three different methods regarding food intake—each aiming to examine different aspects of our objective (Fig. 1). Data from Method A were applied to obtain an overall view on the possible association between food intake and seizure occurrence, while with Method B the exact, predefined preictal intervals were analyzed. Finally, collection of data based on Method C enabled us to exactly measure the duration of food intake-seizure latency.

To determine its possible contributing role in ictogenesis, isolated preictal drinking was analyzed in a similar way. In cases without food or liquid intake during the hour preceding seizures, the latency was estimated based on the ward´s catering protocol and a one-day analysis of habitual food intake. Seizures were characterized based on duration (based on EEG and/or clinical symptoms), seizure type, seizure severity (according to Liverpool Seizure Severity Scale Revisited^6^), propagation of epileptic activity based on scalp EEG recordings (propagation beyond the seizure onset zone, lobe and hemisphere of origin), relations to the sleep/wakefulness cycle (based on EEG recordings) and percentage of antiepileptic drug reduction on the day of seizure occurrence. These parameters were correlated to clinical patient data (gender, age at video-EEG monitoring, age at epilepsy onset, etiology, hemispheric and lobar localization of the epileptic lesion based on imaging and EEG characteristics, type and dosage of antiepileptic medication at admission (normalized as received dose/daily defined dose × 100)^7^.

### Patient groups

Patient groups were created according to the localization of the seizure onset zone, which were classified as temporal, extratemporal, multilobar or generalized. Multilobar seizures were either characterized by an extended epileptic activity on the surface-EEG in non-lesional patients, or based on lesions extending over multiple lobes. Patients with multilobar EEG or lesional involvement onset were merged with patients of a temporal lobe seizure onset zone in case either a localized temporal seizure onset pattern or a clear temporal lesion was present. Further on this merged group will be referred to as “temporal lobe epilepsy” group. Due to the small number of cases, generalized epilepsy patients and patients with hypothalamic hamartoma were excluded from the comparative statistical analyses and are reported separately.

Rates of seizure occurrence in temporal relation with food intake were grouped as: (1) 0%, (2) 1–49%, (3) 50–90% and (4) > 90% depending on the percentage of seizures preceded by food intake within one hour^8,9^.

### Statistical analyses

For general sample characteristics, descriptive statistical methods were applied. For group comparisons, Mann–Whitney, Kruskal–Wallis and chi^2^ tests were used. To analyze relations between food and liquid consumption and seizure onset zone, a generalized estimating equation model was designed, the seizure onset zone (temporal or extratemporal) being the dependent variable. To determine the strength of the association between food intake and seizure occurrence, odds ratios were calculated. Furthermore, a subgroup analysis was also carried out to examine a possible gender difference in this relation. Spearman’s rho correlation was used for continuous variables.

### Ethics

All experimental protocol of the analysis performed was part of seizure prediction studies approved by the Institutional Review Board of the Albert Ludwigs University of Freiburg. Informed consent to the analysis of video EEG recordings was obtained from all patients, or, if participants were under 18, from a parent or legal guardian. All methods were carried out in accordance with relevant guidelines and regulations.

## Results

In our sample the median age was 26 years (r: 1–76; IQR: 28), median age at epilepsy onset was 12 years (r: 0–60; IQR: 17), the median degree of antiepileptic drug dose reduction was 60% (r: − 133–476; IQR: 138). Monitoring the habitual food and liquid consumption, patients ate on average 3 times a day (M: 3; r: 0–8; IQR: 2) and drank on average 5 times a day (M: 5; r: 1–7; IQR: 2). General characteristics of the different patient groups and seizures are presented in Table 1 (additional data are given in Supplement 1). Patients with temporal lobe epilepsy (Group 1) were of higher age at monitoring (*p* = 0.006), higher age at epilepsy onset (*p* = 0.001) and had a higher degree of antiepileptic medication reduction (*p* = 0.002) compared to patients with extratemporal lobe epilepsy (Group 2). In contrast, there was no significant difference in habitual food and liquid consumption (*p* = 0.81 and *p* = 0.54, respectively) and sleep characteristics (*p* = 0.27) between Group 1 and Group 2.

**Table 1:** General characteristics of patients and seizures.

	Temporal	Multilobar	Extratemporal	Generalized	Hypothalamic hamartoma
	Group 1		Group 2	Group 3	Group 4
**Data on patients** **Total number of patients: 100**					
Number of patients	46	10	31	8	5
Gender	14 male (30.4%)	8 male (80%)	13 male (41.9%)	4 male (50%)	2 male (40%)
Age at monitoring (y)	37.7 ± 17.2	15.1 ± 15.2	22.2 ± 14.2	19.8 ± 11.5	11.6 ± 8.9
Age at epilepsy onset (y)	22.1 ± 15.0	8.0 ± 10.4	8.6 ± 7.1	15 ± 10.5	4.0 ± 4.8
**Data on seizures** **Total number of seizures: 592**					
Number of seizures	290 (49.0%)	51 (10.5%)	160 (27.0%)	62 (8.6%)	29 (4.9%)
Food intake 60 min before seizure	73 (25.2%)	20 (39.2%)	16 (10%)	18 (29%)	13 (44.8%)
Drinking 60 min before seizure	122 (42.1%)	24 (47.1%)	31 (19.4%)	28 (45.2%)	12 (41.4%)
Food intake latency (min)	M: 247.5 r: 0–841 IQR: 414	M: 87 r: 0–827 IQR: 175	M: 329.5 r: 2–818 IQR: 331	M: 144.5 r: 1–706 IQR: 262	M: 68 r: 0.5–635 IQR: 241
Drinking latency (min)	M: 80.5 r: 0–841 IQR: 383	M: 61 r: 0–827 IQR: 96	M: 315.5 r: 2–818 IQR: 177	M: 61 r: 0.5–706 IQR: 188	M: 61 r: 0–635 IQR: 208
Seizure severity	M: 21 r: 0–78 IQR: 33	M: 18 r: 0–69 IQR: 26.5	M: 18 r: 0–71 IQR: 10	M: 2 r: 2–265 IQR: 60.5	M: 9 r: 0–68 IQR: 3
AED reduction (%)	M: 117* r: − 17–484 IQR: 150	M: 15 r: − 66–395 IQR: 66	M: 15 r: − 133–233 IQR: 83	M: 63.5 r: 0–200 IQR: 100	M: 60 r: 36–180 IQR: 119

**AED**: antiepileptic drug; **IQR**: interquartile range; **M**: median; **min**: minutes; **r**: range; **sec**: seconds; **y**: years.

*AED reduction values > 100% may appear in patients on polytherapy in whom more than one AED was reduced.

### Seizures preceded by food intake or drinking in light of the seizure onset zone

Table 2 summarizes general patient characteristics according to the rate of food intake-associated seizures.

**Table 2:** Characteristics of patients according to the rate of eating related seizures.

	0%	1–49%	50–89%	≥ 90%
Number of patients	47	33	20	0
Gender	16 male (34%)	13 male (39.4%)	12 male (60%)	–
Age at monitoring (y)	31.3 ± 19.1	30.9 ± 15.9	14.9 ± 11.1	–
Age at epilepsy onset (y)	15.8 ± 14.8	18.3 ± 13.1	7.4 ± 7.1	–
Type of epilepsy	Temporal 18 Extratemporal 23 Multilobar 2 Generalized 2 HH 2	Temporal 21 Extratemporal 4 Multilobar 4 Generalized 3 HH 1	Temporal 7 Extratemporal 4 Multilobar 4 Generalized 3 HH 2	–
Non-lesional epilepsy	17 (36.2%)	5 (15.2%)	5 (25%)	–
Side of the seizure onset zone	Right 15 Left 13 UNDET 15	Right 12 Left 12 UNDET 5	Right 6 Left 3 UNDET 7	–

**HH**: hypothalamus hamartoma; **UNDET**: undetermined; **y**: years.

None of the patients included in this sample did fulfill strict criteria of eating reflex epilepsy^9^. However, in 53% of the total patient population seizures were preceded by food intake within one hour at least once, and 24% of seizures were preceded by food intake in the preictal 1 h period. Food intake was present in 105, 72, 52, 31 and 25 cases in the 45, 30, 15, 5 and 1-min long preictal subintervals, respectively. In 22 seizures, food intake extended into the ictal period.

**Figure 2:**
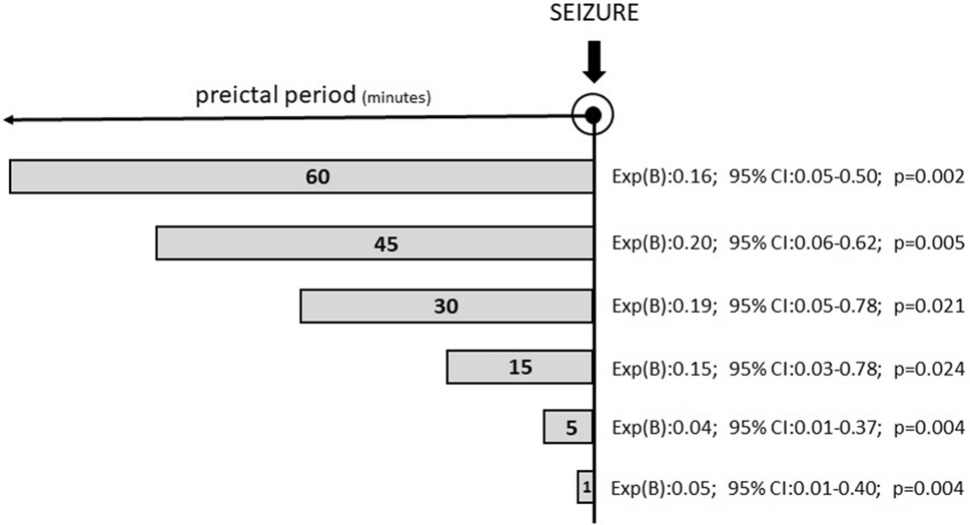
Results of the generalized estimating equations model regarding the effect of eating—subinterval analysis according to Method A.

Seizures involving the temporal lobe occurred more frequently food intake-associated than those of extratemporal origin with food intake in the 60 min-long pre-seizure interval (73/290 in Group 1 vs. 16/160 in Group 2). The odds ratio for seizure occurrence after food intake within 60 min was 2.41 (95% CI 1.63–3.56; *p* < 0.001) in Group 1 and 1.52 (95% CI 0.94–2.47; *p* = 0.88) in Group 2. This association was also detected with the analysis of Method A (Fig. 2). The odds ratio for seizure occurrence was 1.582 (95% CI 1.04–2.42; *p* = 0.04) in Group 1 and 0.52 (95% CI 0.24–1.12; *p* = 0.13) in Group 2 after midday food intake.

In the course of the Method B subinterval analysis, the association of food intake and temporal lobe seizure onset zone could be detected *already* 45–60 min preictally (Exp(B) = 0.199; 95% CI for Exp(B): 0.064–0.620; *p* = 0.005). According to the results of the analysis with Method C, the latency between food intake and seizure onset was shorter in temporal seizures (M: 247, r: 0–841, and M: 329.5, r: 2–818, respectively) (Table 3.) To determine its possible contributing role in ictogenesis, drinking-seizure latency was further added to this model, but isolated liquid consumption showed no seizure triggering effect (Table 3).

**Table 3:** Results of the generalized estimating equations model regarding the effect of eating—food intake-seizure and drinking-seizure latency (Method C).

	Exp(B)	95% CI for Exp(B)	Significance
**A**			
Gender	0.355	0.081–1.561	0.170
Age	1.021	0.972–1.073	0.408
Age at epilepsy onset	0.892	0.830–0.959	0.002
AED reduction	0.990	0.983–0.997	0.004
Food intake-seizure latency	1.002	1.001–1.004	**0.005**
**B**			
Gender	0.367	0.083–1.617	0.185
Age	1.021	0.974–1.071	0.392
Age at epilepsy onset	0.893	0.833–0.959	0.002
AED reduction	0.990	0.983–0.997	0.005
Food intake-seizure latency	1.002	1.001–1.003	**0.043**
Drinking-seizure latency	1.015	0.997–1.033	**0.093**

**AED**: antiepileptic drug; **CI**: confidence interval.

**Figure 3:**
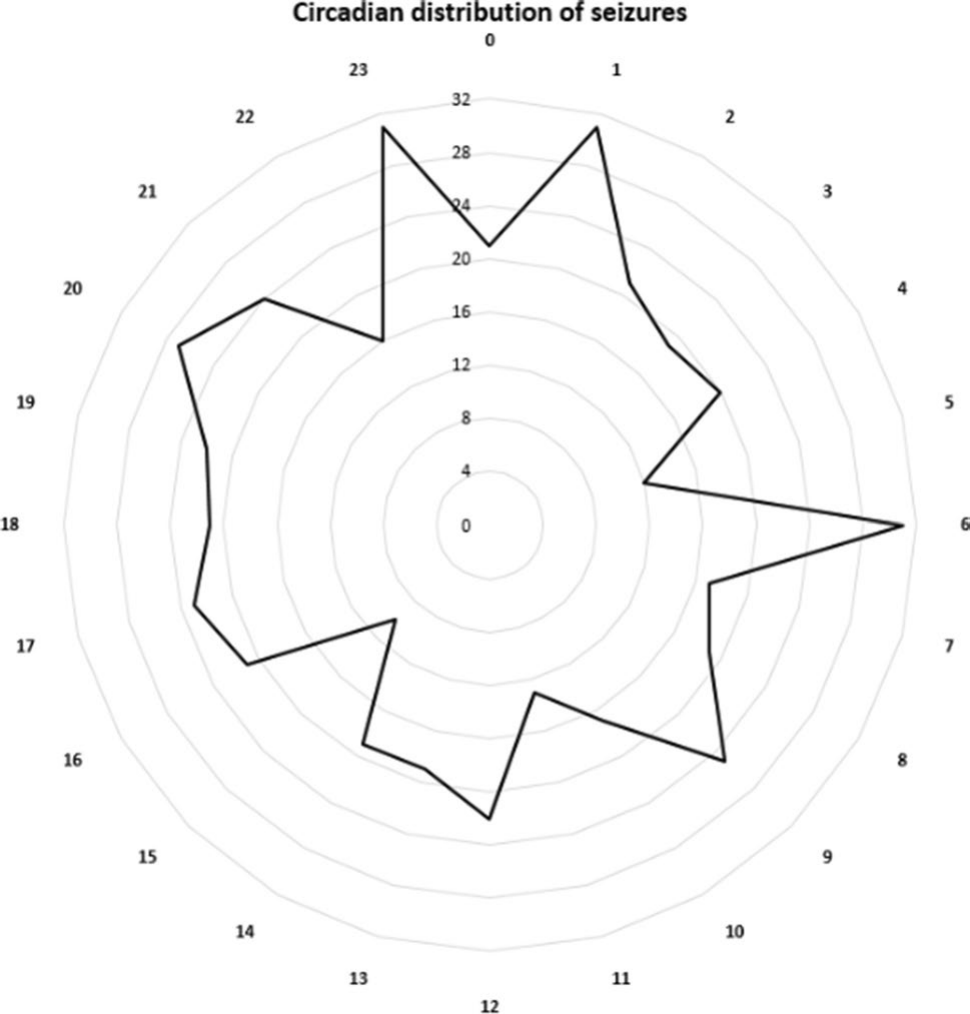
Circadian distribution of seizures.

To determine the possible confounder role of the sleep–wake cycle, the sleep or awake state in which the seizures occurred were also added to the model; and the association between food intake-seizure latency and the seizure onset zone still remained significant (Exp(B) = 1.004; 95% CI for Exp(B): 1.001–1.007; *p* = 0.003). The circadian distribution of seizures are presented in Fig. 3. In patients with hypothalamic hamartoma (Group 4), food intake-associated seizures were even more frequent with 45%. From the 62 seizures of 8 patients with generalized epilepsy (Group 3) with genetic origin 29% occurred within one hour following food intake.

The circle outline timescale is set to hours. The radius represents frequency.

Analyzing male and female patients separately, a male preponderance was detected in patients with food intake-associated seizures (Table 4).

**Table 4:** Results of the generalized estimating equations model regarding the gender differences of the effect of eating (Method C).

	Exp(B)	95% CI for Exp(B)	Significance
**Male (number of seizures: 186)**			
Age	1.052	0.971–1.140	0.211
Age at epilepsy onset	0.859	0.748–0.986	0.030
AED reduction	0.998	0.988–1.009	0.742
Food intake-seizure latency	1.063	1.005–1.125	**0.033**
**Female (number of seizures: 315)**			
Age	0.993	0.936–1.054	0.828
Age at epilepsy onset	0.901	0.818–0.993	0.828
AED reduction	0.985	0.975–0.995	0.003
Food intake-seizure latency	1.036	0.984–1.089	**0.177**

**AED**: antiepileptic drug; **CI**: confidence interval.

Statistical evaluation using Mann–Whitney U test in Group 1 confirmed male preponderance also when comparing the mean eating-seizure latency of each patient (224 min vs. 294 min in males and females, respectively; *p* = 0.028). No such difference could be detected between males and females in Group 2 (397 min vs. 366 min in males and females, respectively; *p* = 1.000).

### Characteristics of seizures associated to food intake

Shorter food intake-seizure latency was associated with less severe seizures (r_s_ = 0.35; *p* = 0.001). This correlation was present both, in Group 1 (LSSS M: 21, r: 0–78) and Group 2 (LSSS M: 18, r: 0–71) (*p* < 0.001 and *p* < 0.001, respectively). In patients of any seizure origin, food intake-seizure latency was shorter in seizures with preserved awareness than in seizures with impaired awareness and focal to tonic clonic seizures (*p* < 0.001). In contrast, there was no significant correlation between food intake-seizure latency and seizure duration (EEG: r_s_ = 0.07; *p* = 0.12; clinical: r_s_ = 0.02; *p* = 0.93), seizure propagation from the seizure onset zone (*p* = 0.07) or seizure propagation from the seizure onset lobe (*p* = 0.08). Furthermore, contralateral spread occurred significantly more frequent in seizures not triggered by food intake. (*p* = 0.025).

## Discussion

To our best knowledge, this is the first study investigating food intake as a seizure precipitant based on objective data, i.e. continuous video-EEG monitoring. Our data provide evidence that food intake is a significant seizure precipitant in patients with focal epilepsy of temporal origin, even if none of the patients fulfilled the old concept of reflex epilepsy. The strong association of food intake and subsequent seizure occurrence modifies concepts of “spontaneous” ictogenesis und supports the notion of non-random seizure occurrence related to exogenous modifiers of brain excitability.

### Seizure facilitating and precipitating/triggering factors: the twilight of the general concept of eating reflex epilepsy

It is currently assumed that ictogenesis is a result of the interplay of two main factors. Endogenous facilitating factors (e.g. the presence of gene mutations or epileptogenic brain lesions) causing fluctuations of baseline seizure threshold, render the nervous system more prone to generate epileptic seizures in response to the so-called precipitating or triggering factors, which exhibit a short-term and direct effect on seizure occurrence^10^. Triggering/precipitating factors can be categorized as either extrinsic (e.g. flashes of light, sounds, hot water) or intrinsic (e.g. cognition, emotional responses)^10^. If one such factor repeatedly and consistently triggers seizures, it is classified as a reflex seizure, where a strong temporal connection can be established between the triggering factor and the seizure occurrence: e.g. startle, music, photic, reading, hot water or—in this case—eating induced seizures^10^. Eating reflex epilepsy in its strict sense has a prevalence between 0.006 and 0.067%^8,11^ and is characterized by seizures occurring consistently during or immediately after the consumption of food, as the result of a dynamic interplay between an underlying hyperexcitable epileptic network and the food intake related possible triggering factors (e.g. mastication, tasting, swallowing, gastric distension, emotional responses). By definition, triggered seizures make up all or nearly all seizures of these patients. In 1945 Allen^12^, than later Boudouresques and Gastaut reported on patients who experienced prandial and postprandial seizures^13^. Since then, several case reports and case series have been published^8,11,14,15^ however, with inconsistent results regarding the condition’s characteristics, and conflicting opinions on the overall nature and pathophysiology of the disease. Characteristics considered to be typical of eating reflex epilepsy are (1) seizures with preserved or impaired awareness of (2) temporal lobe origin, appearing on the ground of a (3) non-lesional, (4) therapy resistant epilepsy with a (5) male predominance, showing a (6) strong temporal connection to food consumption^11,16,17^. However, epileptic spasms^18–20^, extratemporal seizure onset zone^8^, a variety of brain lesions and female gender have also been associated with this condition^14,21^. Moreover, no clear definition of eating reflex epilepsy has been established yet, i.e. the rate of eating reflex seizures to all seizures and the latency between eating and seizure occurrence have not been precisely defined^8,9,22^. In recent years, the concept of separate reflex epilepsies has been challenged due to growing evidence towards a continuum between spontaneous and reflex seizures and intrinsic and extrinsic factors modulating seizure propensity^10,23^.

### The possible role of the temporolimbic hyperexcitable network in the occurrence of food intake-associated seizures

Not a single patient of this sample of 100 patients fulfilled criteria for eating reflex epilepsy in that > 90% of the recorded seizures are preceded by food consumption^8^. However, we detected a clear association between food intake and seizures of temporal lobe origin: the risk of seizure occurrence is twice as high in the 60 min-long postprandial period. Patients with focal epilepsy of temporal lobe origin showed preictal food intake more often than those of extratemporal origin: food intake preceded temporal lobe seizures 15% more often than those of extratemporal origin (25% in temporal vs. 10% in extratemporal seizures) independently of the patients’ habitual food consumption, which showed no difference regarding the frequency of food intake. Latencies between food intake and seizure occurrence were shorter in patients with temporal lobe seizures with a decreasing probability of temporal lobe seizure occurrence by 0.2% per minute. Of interest, food intake did not only immediately trigger seizures but was already associated with an increased seizure occurrence probability 45–60 min preictally, which suggests that not only the direct effects of food intake play a role in seizure occurrence. Analyses of circadian influences on seizure occurrence have noted an increase in seizure probability during early morning hours and in the evening^3^. We have thus analyzed the timing of seizures in our patient cohort with particular view on seizures occurring at midday food intake, which also showed increased odds for temporal lobe seizure after lunch.

Based on our results we suppose that food intake-associated seizures are caused by activation of central processing of alimentation based on an interplay with the hyperexcitable temporolimbic network involved both in the physiological aspects of alimentation and in seizure generation. Functional MRI studies clearly identified the hypothalamus, insula, amygdala and hippocampus as central structures of the appetitive network^24–26^. A possible role of the temporolimbic network is also corroborated by the particularly high rate of food intake-associated seizures (45%) in patients with hypothalamic hamartoma. This brain region acts as one of the main central processing areas in food consumption^27^. Based on our results, proictal effects of food intake may activate brain networks in several ways.

Mastication may act as a direct trigger, as isolated liquid intake showed no triggering effect. Interestingly, mastication has been shown to exert considerable electrical fields modulating temporal neocortical activity in epileptic patients undergoing intracranial EEG monitoring^28^. In a mice model, in this line, antimuscarinic-induced convulsions in mice did not occur in response to receiving liquid, as opposed to solid food^22^. Conversely, temporal networks are involved in mastication, and repetitive electrical stimulation of the amygdala triggers masticatory jaw movements^29^. Longer lasting proictal effects of food intake suggest that alimentary networks beyond mastication contribute to the triggering of seizures^22^, including olfactory, gustatory, interoceptive (e.g. gastric distension) processing, neurohumoral (ghrelin related) and emotional responses within the temporolimbic network^30–32^. In summary, the results suggest both, fast-acting or slow-acting precipitating effects of food intake on temporal seizure generation^10^. Whereas mastication may play a role as a fast-acting trigger, more complex activation of alimentary pattern generators, sensory input, emotional responses or hormonal changes may exert a slower modulatory effect on seizure occurrence.

In our patient sample, seizure triggering effect of food intake became significant only in males, which implies a gender-depending susceptibility of external seizure trigger factors. Similar to this finding, also a systematic review studying 378 patients with eating reflex epilepsy showed a male preponderance with 72% of reported patients being males^16^. In our cohort, shorter food intake-seizure latency was associated with less severe seizures and preserved awareness. Moreover, food intake induced more focalized seizures compared to seizures without a close food-trigger, again pointing to a local network activating effect.

Although the predominance of non-lesional epilepsy has been reported in eating reflex epilepsy^8^, it has been previously suggested that structural changes, by acting as a switch between physiological and abnormal brain activity rendering the brain more susceptible to focal reflex seizures; accordingly, eating reflex seizures have also been reported in focal epilepsies of structural origin, such as cortical malformations or hypoxic cerebral lesions^23,33–35^. Our results correspond to this theory: in patients with no food intake-associated seizures the rate of non-lesional epilepsy was 36.2%, while in those where 50–89% of the seizures were food intake associated, only 25% were be MRI negative (Table 2).

Aside from focal epileptogenesis, also genetically determined hyperexcitability may be associated with food intake-associated seizures^9^, e.g. also in specific syndromes like Rett syndrome^36^ and SYNGAP 1 gene mutations^37,38^. Our patient sample was too small to analyze genetic effects in detail but also found food-intake associated seizures in 29% of the seizures of genetic, generalized epilepsy.

## Limitations

We limited the detailed analysis to a preictal period of 60 min, leaving open more temporally extended effects of food intake on seizure occurrence. Possible effects of blood sugar level fluctuations related to food intake should be studied prospectively based on the findings of this study. To further elucidate relevant mechanisms involved in food-triggering of seizures, additional biomarkers like hormonal levels may contribute a further understanding of mechanisms involved in seizure-triggering. We did not relate findings to drug levels as these were not available at the timing of each seizure, but limited our analyses to the daily defined and the actual dose of medication. The analysis was furthermore limited to focal seizures and leaves open the role of food intake in generalized epilepsy.

## Conclusion

This retrospective video-EEG based study for the first time demonstrates food intake as a seizure-triggering factor in patients beyond the classical reflex epilepsy, characterizing the region of seizure onset, identifying a gender effect, and assessing the clinical manifestation of seizures occurring in the context of food intake. We found that food intake-associated seizures are characteristic for temporal lobe epilepsy, with significant male predominance. Analysis of an extended, 60 min-long period preceding seizures reveals that food consumption carries not only an immediate but also a delayed seizure triggering effect, which can be detected at least for a period of up to one hour preictally. Moreover, there proved to be an association between food intake-seizure latency, seizure semiology and seizure severity, which not only carries interesting hints for clinical practice but could also supports recently emerging concepts of ictogenesis in temporal and more extended brain networks.

## Supplementary Information

Supplementary Table.

## Data Availability

The datasets generated during and analyzed during the current study are available from the corresponding author on reasonable request.
