# Supplementary material for: Food intake precipitates seizures in temporal lobe epilepsy
Source: Sci Rep. 2021 Aug 13;11:16515. doi: 10.1038/s41598-021-96106-z (PMC8363749; doi:10.1038/s41598-021-96106-z)
Supplement: Supplementary file 1 — Supplementary Table. [file 41598_2021_96106_MOESM1_ESM.docx]

**Supplement 1**

**Table 1 - extended**

|  | **Temporal** | **Extratemporal** | **Multilobar** | **Generalized** | **Hypothalamic hamartoma** |
| --- | --- | --- | --- | --- | --- |
| **DATA ON PATIENTS**  **TOTAL NUMBER OF PATIENTS: 100** | | | | | |
| Number of patients | 46 | 31 | 10 | 8 | 5 |
| Gender | 14 male (30.4%) | 13 male (41.9%) | 4 male (40%) | 8 male (100%) | 2 male (40%) |
| Age at monitoring (y) | 37.7±17.2 | 22.2±14.2 | 15.1±15.2 | 19.8±11.5 | 11.6±8.9 |
| Age at epilepsy onset (y) | 22.1±15.0 | 8.6±7.1 | 8.0±10.4 | 15±10.5 | 4.0±4.8 |
| Etiology | HS/HA 15 (32.6%)  MCD 3 (6.5%)  tumor 7 (15.2%)  non-lesional 16 (34.8%)  non specific 5 (10.9%) | MCD 10 (32.3%)  tumor 2 (6.5%)  tuberous sclerosis 2 (6.5%)  non specific 9 (29.0%)  non-lesional 8 (25.8%) | MCD 2 (20.0%)  tuberous sclerosis 2 (20.0%)  Sturge-Weber syndrome 1 (10.0%)  non-lesional 3 (30.0%)  non specific 2 (20.0%) | IGE 7 (87.5%)  Sotos-syndrome 1 (12.5%) |  |
| Side of SOZ | left 21  right 20  UNDET 5 | left 6  right 10  UNDET 15 | left 1  right 2  UNDET 7 |  |  |
| **DATA ON SEIZURES**  **TOTAL NUMBER OF SEIZURES: 592** | | | | | |
| Number of seizures | 290 (49.0%) | 160 (27.0%) | 51 (10.5%) | 62 (8.6%) | 29 (4.9%) |
| Food intake 60 min before seizure | 73 (25.2%) | 16 (10%) | 20 (39.2%) | 18 (29%) | 13 (44.8%) |
| Drinking 60 min before seizure | 122 (42.1%) | 31 (19.4%) | 24 (47.1%) | 28 (45.2%) | 12 (41.4%) |
| Food intake latency (min) | M: 247,5  r: 0-841  IQR: 414 | M:329,5  r: 2-818  IQR: 331 | M: 87  r: 0-827  IQR: 175 | M: 144,5  r: 1-706  IQR: 262 | M: 68  r: 0,5-635  IQR: 241 |
| Drinking latency (min) | M: 80,5  r: 0-841  IQR: 383 | M:315,5  r: 2-818  IQR: 177 | M: 61  r: 0-827  IQR: 96 | M: 61  r: 0.5-706  IQR: 188 | M: 61  r: 0-635  IQR: 208 |
| Seizure severity | M: 21  r: 0-78  IQR: 33 | M: 18  r: 0-71  IQR: 10 | M: 18  r: 0-69  IQR: 26.5 | M: 2  r: 2-265  IQR: 60.5 | M: 9  r: 0-68  IQR: 3 |
| Seizure type | SPA 76 (26.2%)  SIA 168 (57.9%)  FTCS 33 (11.4%)  UNDET 13 (4.5%) | SPA 55 (34.4%)  SIA 62 (38.8%)  FTCS 17 (10.6%)  UNDET 26 (16.3%) | SPA 10 (19.6%)  SIA 30 (58.8%)  FTCS 4 (7.8%)  UNDET 7 (13.7%) |  | SPA 17 (58.6%)  SIA 4 (13.8%)  FTCS 0  UNDET 8 (27.6%) |
| Seizure duration (EEG) (sec) | M: 52  r: 2-810  IQR: 63 | M: 32  r: 1-1408  IQR: 57 | M: 59  r: 4-928  IQR: 85 | M: 2  r: 1-671  IQR: 14.5 | M: 31  r: 19-118  IQR: 51 |
| Seizure duration (Clinical) (sec) | M: 66.5  r: 1-773  IQR: 63 | M: 23  r: 1-928  IQR: 45 | M: 71  r: 11-819  IQR: 93 | M: 2  r: 1-121  IQR: 24 | M: 52.5  r: 10-124  IQR: 56 |
| Propagation from SOZ/SOL/SOH | SOZ 79.5%  SOL 78.6%  SOH 58% | SOZ 66%  SOL 66%  SOH 57% | SOZ 70.3%  SOL 65.6%  SOH 40% |  |  |
| Propagation speed from SOZ/SOL/SOH (sec) | SOZ  M: 4; r: 0.5-252; IQR: 12  SOL  M: 4; r: 0.5-252; IQR: 12  SOH  M: 9; r: 0,5-501; IQR: 18.5 | SOZ  M: 3; r: 0.5-54; IQR: 6  SOL  M: 3; r: 0.5-54; IQR: 8  SOH  M: 4; r: 0.5-50; IQR: 11 | SOZ  M: 2; r: 0.5-36; IQR: 3.5  SOL  M: 2.5; r: 0.5-36; IQR: 4.5  SOH  M: 4; r: 0.5-239; IQR: 13 |  |  |
| Sleeping 60 min before seizure | 70 (24.1%) | 46 (28.8%) | 16 (31.4%) | 17 (27.4%) | 4 (13.8%) |
| AED reduction (%) | M: 117*  r: -17-484  IQR: 150 | M: 15  r: -133-233  IQR: 83 | M: 15  r: -66-395  IQR: 66 | M: 63.5  r: 0-200  IQR: 100 | M: 60  r: 36-180  IQR: 119 |

**AED**: antiepileptic drug; **EEG**: electroencephalography; **FTCS**: focal to tonic clonic seizure; **HA**: hippocampal atrophy; **HS**: hippocampal sclerosis; **IGE**: idiopathic generalized epilepsy; **IQR**: interquartile range; **M**: median; **MCD**: malformation of cortical development; **min**: minutes; **r**: range; **sec**: seconds; **SIA**: seizure with impaired awareness; **SOH**: seizure onset hemisphere; **SOL**: seizure onset lobe; **SOZ**: seizure onset zone; **SPA**: seizure with preserved awareness; **UNDET**: undetermined; **y**: years

*AED reduction values >100% may appear in patients on polytherapy in whom more than one AED was reduced
